# Rotterdam Oncology Documentation (RONCDOC) – a high-quality data warehouse and tissue collection for head and neck cancer

**DOI:** 10.1186/s12885-025-14100-4

**Published:** 2025-04-25

**Authors:** Arta Hoesseini, Emilie A. C. Dronkers, Eveline Dieleman, Maria J. De Herdt, Marjan. H. Wieringa, Marie-Louise F. van Velthuysen, Marinella P. J. Offerman, Aniel Sewnaik, Dominiek Monserez, Stijn Keereweer, Jose A. U. Hardillo, Robert Jan Baatenburg de Jong

**Affiliations:** 1 Department of Otorhinolaryngology and Head and Neck Surgery, Erasmus MC Cancer Institute, Erasmus University Medical Center, Dr. Molewaterplein 40, Rotterdam, 3015 GD The Netherlands; 2 Department of Otorhinolaryngology and Head and Neck Surgery, Leiden University Medical Centre, Leiden, The Netherlands; 3 Department of Pathology, Erasmus MC, Erasmus University Medical Center, Rotterdam, The Netherlands; 4 Department of Education and Research, Elisabeth TweeSteden Hospital (ETZ), Tilburg, The Netherlands

**Keywords:** Head and neck cancer, Oncology, Database, Cohort study

## Abstract

**Background:**

Every year, almost 900.000 people are diagnosed with head and neck cancer (HNC) worldwide. HNC contains many different subsites and a large variability in tumor biology. This often results in small and/or heterogeneous study populations. Developing overarching databases is an efficient solution to collect and analyze data of these smaller subsets of patients and to facilitate data sharing among research groups. The few existing large databases often include only basic characteristics. In addition, hospital-based cohorts that include more variables are often not collected consecutively, resulting in selection bias. Therefore, we established a hospital-based cancer registry system “Rotterdam Oncology Documentation” (RONCDOC), a complete and consecutive data warehouse and tissue collection for HNC, directly registered at the source. The primary aim of this paper is to report on our data collection protocol in order to make the RONCDOC data accessible and reusable for other researchers, and to offer a blue print to other consortia planning to establish their own data warehouse.

**Methods:**

Data collected in the Netherlands Cancer Registry (NCR) of patients with HNC were obtained from the Netherlands comprehensive cancer organization (IKNL) and merged with corresponding data from the electronic patient file (EPF). The data were manually verified using the EPF, and enriched with additional variables from the EPF according to an extensive data entry protocol. Furthermore, a comprehensive validation protocol was developed to guarantee the quality of the data. Tissue microarrays (TMAs) were constructed from resection specimens of patients with primary oral squamous cell carcinoma.

**Conclusion:**

With RONCDOC, we have established a consecutive and high-quality data warehouse for HNC. This paper outlines the essential steps for establishing such a data warehouse, offering a blueprint for other consortia.

**Trial registration:**

This study was approved by the ethics committee of the Erasmus Medical Center (MEC-2016–751).

**Supplementary Information:**

The online version contains supplementary material available at 10.1186/s12885-025-14100-4.

## Background

Researchers often acquire similar data and store them in separate data sets making it difficult to reuse data for purposes other than the initial research question. This does not only occur among different centers but also within one clinical center, within different disciplines or even within a research group. It is valuable, cost-effective and more efficient for novel research projects, when researchers would be able to combine these datasets. This facilitates data sharing among research groups. In addition, an overarching database facilitates collaboration in multi center studies. Almost 900.000 people per year are diagnosed with HNC worldwide [1]. More than 90% of all HNCs are squamous cell carcinomas [2]. HNC represents a variety of (squamous cell) tumors originating from the lip, oral cavity, oropharynx, hypopharynx, larynx, (sino)nasal cavity, nasopharynx and salivary glands. In the Netherlands, around 3000 new patients are diagnosed with HNC annually [3]. A patchwork of different subsites with different biology disperses the already relatively small numbers of HNC patients per year. This often results in small and/or heterogeneous study populations. The few existing large population based cohorts in the world containing HNC include TNM classification, tumor location and survival data but often lack more patient and tumor specific variables such as smoking behavior, comorbidity, histopathology and/or molecular features [4–7]. Hospital based cohorts that do include these variables are often not collected consecutively resulting in selection bias. Therefore, we established the hospital-based cancer registry system “Rotterdam Oncology Documentation” (RONCDOC), a complete, high-quality and consecutive data warehouse including clinical, diagnostic and therapeutic data and tissue collection for HNC, directly registered at the source. The primary aim of this paper is to report on our data collection protocol in order to make the RONCDOC data accessible and reusable for other researchers, and to offer a blueprint to other consortia planning to establish their own data warehouse.

## Construct and content

### Design and setting

**Fig. 1:**
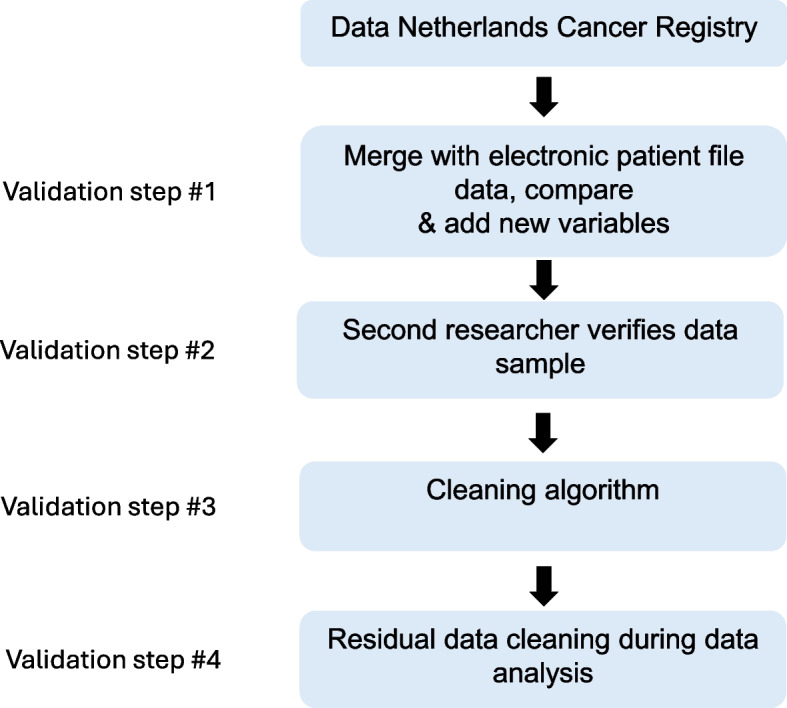
RONCDOC design and validation steps

Data collected in the Netherlands Cancer Registry (NCR) of patients with HNC were obtained from the Netherlands comprehensive cancer organization (IKNL). Information on every patient with cancer in the Netherlands is recorded in the NCR. In general, data collected by the IKNL includes variables like sex, age, cTNM, pTNM, tumor morphology, tumor topography, and date of death. Data from the IKNL are retrospectively collected by trained data managers. In contrast, data in the Electronic Patient File (EPF) contains more specific patient and tumor data and is directly registered at the source. Since 2016, our institution has implemented the use of an individual tumor board form for each new head and neck tumor that includes several mandatory variables beyond the TNM classification, such as comorbidities, height, weight, smoking status, WHO performance status, and alcohol consumption. This facilitates standardized data collection. All variables are initially registered in the EPF and subsequently in the tumor board form. Therefore, the EPF serves as the primary data source. In RONCDOC, the consecutive NCR data of patients with HNC were first merged with corresponding clinical data from the EPF of the Erasmus Medical Center Cancer Institute (see Fig. 1, validation step 1). These variables were manually verified by trained medical students using the EPF, and enriched with additional variables from the EPF according to an extensive data entry protocol. The data entry protocol provides descriptions of all variables listed in Table 1, along with examples. The complete data dictionary and data entry protocol are added as a supplementary file. All medical students were extensively trained by senior medical students, senior researchers, and medical doctors on how to use the data entry protocol. When data of the NCR did not correspond with the data in the EPF, the latter was considered superior. In case of doubt, selected cases were discussed in the research staff. During the second validation step, students verified data samples of each other (internal control). Around 10% of the total processed data by one student was checked by another student or by a senior researcher. If a variable showed deviating results, the matter was examined, and the data were manually adjusted where necessary. Hereafter, data were verified again using a cleaning algorithm (validation step 3): for each variable, a separate “cleaning syntax” was created in SPSS in order to review the data and adjust if necessary. Categorical values were first checked for correct entry. Some missing data were verified again in the EPF by a different student or senior researcher and adjusted if necessary. Any additional irregularities identified were addressed similarly. Finally, during the analyses of various research projects, any remaining data cleaning was performed if necessary (step 4). For example, when a variable initially considered missing was later identified in the EPF by a different researcher. All baseline variables were scored according to their outcome at the time of diagnosis, and a log was kept in which members of the research team entered the data. In addition, a log was kept of all changes made.

**Table 1:** Overview of the variables available in RONCDOC in 2025

Variable	Category
**Patient**	
Sex	Female/male
Date of birth	Date
Deceased	Yes/no
Final date of follow-up/date of death	Date
Cause of death	Due to HNC/not due to HNC/unknown/not applicable
Smoking	Yes/no/former
Pack years	
Alcohol consumption	Yes/no/former
No. alcohol units per week	
Adult Comorbidity Evaluation 27	Subscales & total scale
Weight	Kilogram (1 kg = 2.20 pounds)
Weight loss in the past six months	Kilogram (1 kg = 2.20 pounds)
Height	Centimeter (1 cm = 0.39 inches)
Body Mass Index	
WHO performance status (Eastern Cooperative Oncology Group score)	0/1/2/3/4
Anemia	Yes/no, cutoff male < 8.5 mmol/L, cutoff female < 7.5 mmol/L (1 mmol/L = 1.61 g/dL)
Heart valve disease	Yes/no
Marital status	Married / in a durable relationship or single / widowed
Prior malignancy	Yes/no
Localization prior malignancy	Lung/breast/bowel/prostate/hematologic/head and neck/other
Year of diagnosis of prior malignancy	Year
Treatment for prior malignancy?	Yes/no
Type of treatment for prior malignancy	None/radiotherapy/chemoradiation/surgery/surgery & radiotherapy/chemoradiation & surgery/chemotherapy/surgery & chemotherapy
**Tumor**	
Date of diagnosis	Date
Tumor no in chronologic order	1, 2, 3, 4, 5, etc
Primary or recurrent tumor	First primary/second primary/etc First recurrence/second recurrence/etc
Recurrence related to	First primary/second primary/etc
Synchronous/metachronous tumor	Not applicable/synchronous/metachronous
Tumor topography	Lip/oral cavity/oropharynx/hypopharynx/pharynx NOS^a^/glottic larynx/supraglottic larynx/subglottic larynx/larynx NOS^a^/nasopharynx/unknown primary/salivary glands/nasal cavity/middle ear/sinus/thyroid/skin
Site of tumor	Left/right/middle/bilateral
cTNM	According to the TNM- 7
pTNM	According to the TNM- 7
Tumor morphology	Squamous cell carcinoma/adenocarcinoma/etc
Treatment intention	Curative/palliative because of HNC/palliative because of another tumor/refusal of curative treatment/died before completing diagnostics/unknown
Retropharyngeal nodes	Yes/no
HPV-status (p16 immunoreactivity)	Yes/no
**Treatment**	
Treatment intention	Curative/palliative because of HNC/palliative because of another tumor/refusal of curative treatment/died before completing diagnostics/unknown
Did the patient receive treatment?	Yes/no (patient related)/no (physician related)/died before start treatment
Treatment according to protocol?	Yes/no (patient related)/no (physician related)/died before start treatment
Treatment type no. 1^b^	None/radiotherapy/chemotherapy/surgery with lymph node dissection/surgery without lymph node dissection/lymph node dissection/other
Start date of treatment no. 1	Date
End date of treatment no. 1	Date
Was treatment no. 1 completed?	Yes/no/not applicable/unknown
**Pathology**	
Pathology specimen ID	
Pathology specimen date	Date
Pathology specimen type	Biopsy/resection/etc
Pathology specimen characteristics	Tumor site & location/tumor diameter/no. pathological lymph nodes affected (side & level)/diameter of metastasis in mm (if RLNMs are present)/extranodal extension (if RLNMS are present)/depth of invasion (according to 8 th ed AJCC)/resection margin status/WPOI (intended according to 8 th ed AJCC)/invasion pattern/perineural invasion/lymphovascular invasion/histological grade/tumor infiltrating lymphocytes (intended according to 8 th ed AJCC)

Several free text fields were added between variables (not included in this table). A complete overview of all variables can be found in the data dictionary and data entry protocol

^a^Not Otherwise Specified

^b^For each treatment type a separate column was completed on type, start date, end date and completion (no. 1, no. 2, etc.)

### Study population

Inclusion started from the 1st of January 2006, in correspondence with the transition from paper charts to the EPF. Data of patients treated between the 1st of January 2006 and the 31st of December 2016 has been completed (*N* = 3728). Currently, we are gathering data from 2017 until present. Follow-up time has been repeatedly updated by consulting the Municipal Personal Records Database (MPRD). The final day of follow-up was defined as the final date that the patient was confirmed to be alive (last registered hospital contact) or the date of death.

### Clinical, diagnostic and tumor tissue data collection

Clinical data, diagnostics and tumor tissue data were collected. Table 1 shows an overview of the variables included in RONCDOC. Number of alcohol units per week were scored according to a standardized list: one unit, or 10 g of alcohol is equivalent to 12.5 ml of pure ethanol [8]. The cumulative quantity of smoking was defined in pack-years in which one pack year was equal to one pack of 20 cigarettes smoked per day for one year. If a patient had stopped smoking for ≥ 3 months, he or she was considered as a *former smoker*. If a patient had stopped drinking for ≥ 6 months he or she was considered as a *former drinker*. Comorbidity was scored using the Adult Comorbidity Evaluation- 27 (ACE- 27) which is a 27-item validated comorbidity index covering nine organ systems [9]. Weight loss in kilograms (kg) was defined as weight loss in the six months before diagnosis. The World Health Organization (WHO) performance status, also known as the Eastern Cooperative Oncology Group (ECOG) score, was scored according to the classification published by Oken et al. [10] Marital status was defined as being married or having a durable relationship versus being single or widowed. TNM was staged according to the 7th American Joint Committee on Cancer (AJCC) edition of the TNM classification of malignant tumors [11]. Synchronous tumors were defined as tumors that were diagnosed ≤ 6 months after diagnosis of the index tumor and metachronous tumors >6 months. In case of oropharyngeal tumors, immunohistochemical analysis was performed for tumor suppressor protein p16 (cyclin-dependent kinase 2 A). p16 Positivity was defined as strong and diffuse nuclear and cytoplasmic immunostaining in > 70% of the tumor cells. p16 Positive tumors were considered human papilloma virus (HPV) positive according to the 8th AJCC TNM guideline [12, 13]. Histopathological characteristics were extracted from the pathology reports. Infiltration depth of oral squamous cell carcinoma was assessed according to the 7th edition of the AJCC [11]. The depth of invasion was revised – using scans of hematoxylin and eosin stained (HE) slides representative for the cancer resection specimen – according to the 8 th AJCC edition of the TNM classification of malignant tumors [12, 13]. All revisions were performed in collaboration with a dedicated head and neck pathologist.

### Tissue Microarray production and sample collection

Tissue microarrays (TMAs) were constructed from formalin-fixed paraffin-embedded (FFPE) resection specimens of patients with primary oral squamous cell carcinoma. Patients with simultaneous primary cancers in the head and neck region were excluded from sampling. Prior to the TMAs construction, hematoxylin and eosin (HE) sections – representing the selected cancers – and their corresponding FFPE tissue blocks were collected from the tissue archive of the pathology department. Subsequently, a dedicated head and neck pathologist examined all HE slides with special attention to the following pathological characteristics: cancer type, differentiation grade, depth of invasion, growth pattern, perineural invasion, vasoinvasive growth, extranodal growth and bone invasion and selected vital cancer regions that were properly fixated for coring. Three cores (1.0 mm diameter) were sampled for pT1 - 2 cancers: one from the center and two from the periphery. For pT3 - 4 cancers, four cores (1.0 mm diameter) were sampled: two from the center and two from the periphery. Tissue microarray cores were sampled from donor blocks and positioned in acceptor blocks using the TMA Grand Master (3DHISTECH Ltd.; Budapest; Hungary). Tissues were used according to the “The Code for Proper Secondary Use of Human Tissue” and “The Code of Conduct for the Use of Data in Health Research” as stated by the Federation of Dutch Medical Scientific Societies [14, 15].

### Data storage & coding

Patient data were entered and stored in GEneric Medical Survey Tracker (Gemstracker) [16]. This software package allows data collection and is especially developed for clinical research and quality registrations in healthcare. The software is published under an open-source license and allows coded data collection. RONCDOC data were coded by study ID. FFPE blocks representing the tissue microarray are stored at the department of pathology of the Erasmus MC.

### Data and sample dissemination

The dissemination of RONCDOC data is regulated in the RONCDOC collaboration agreement. A request for data release can be sent to the RONCDOC consortium by submitting a research proposal in a standardized format. The consortium consists of the departments: Otorhinolaryngology and Head and Neck Surgery, Radiotherapy, Plastic and Reconstructive Surgery, Oral and Maxillofacial Surgery, Radiology and Nuclear Medicine, Pathology and Internal Oncology within the Erasmus Medical Center. During quarterly meetings the research proposals are evaluated and discussed. Researchers are encouraged to introduce themselves or a medical student as a new member of the research team when they submit a new research proposal. When additional data are collected in the course of new research projects, these are added to RONCDOC.

## Utility and discussion

RONCDOC has been built to develop a consecutive and high-quality data warehouse and tissue collection for HNC. Extensive validation contributed to a high degree of accuracy and a low risk of bias. This combination of high-quality data with tumor tissue collection can be of high value for future research. The strength of RONCDOC is its completeness in collected variables like general socio-demographic information, detailed information on co-morbidity including ACE-27 scores, detailed treatment information, data on health and lifestyle, follow-up and pathology data. The variables are registered directly at the source and at time of diagnosis of each new primary head and neck tumor. Aside from being comprehensive, RONCDOC is set up as a consecutive database. A consecutive registration has advantages over other registrations as a consecutive design does not have to take selection bias into account.

### Overview of existing HNC databases

To date, there are few other large oncology databases including national registries and international studies that include HNC. Most of these databases mainly cover other cancer types, yet some databases are specifically designed for HNC. Table 2 provides an overview of the various existing databases, including RONCDOC, to the best of our knowledge. Most of the databases were developed to get insight in national cancer care. Although there is a significant overlap in collected variables such as patient demographics, tumor staging and first course of treatment, important differences exist. The National Cancer Database (NCDB) [17, 18] provides data on comorbidities using the Charlson comorbidity index (CCI) while the Surveillance, Epidemiology, and End Results SEER program (SEER) [4, 5] database does not [19]. The NCDB data are hospital based contrary to the population based data in the SEER database meaning that the NCDB only includes data from patients diagnosed or receiving treatment in hospitals accredited by the American College of Surgeons’ Commission on Cancer [18, 19]. Currently, these hospitals represent approximately 30% of all hospitals in the U.S. covering about 70% of all patients newly diagnosed with cancer [17, 18]. The SEER program covers approximately 35% of the U.S. population including data from different geographic areas representative of the demographics of the complete U.S. population [4, 5]. To improve the latter national databases in the U.S., the National Program of Cancer Registries (NPCR) was founded supporting statewide, population based cancer registries in 1992 [20–22]. All these databases consist of a considerable large amount of data presuming to yield national coverage, but are formed retrospectively and in a non-consecutive manner. Another particular large database, NORDCAN, is an international database covering approximately 98% of all cancers diagnosed in the Nordic countries (Denmark, Finland, Iceland, Norway, Sweden, Faroe Islands and Greenland) [6, 7]. On the one hand NORDCAN describes both national and international incidence, prevalence and mortality, on the other hand detailed information about recurrence and co-morbidity is lacking [6, 7]. The Cancer Genome Atlas (TCGA) collected a comprehensive set of data to examine the molecular basis of cancer, including HNC [23]. In the Netherlands, the IKNL provides the NCR, a national cancer registration including many variables on patient and tumor characteristics [24]. In addition to the abovementioned databases, there are few databases that are prospective and specific to HNC. Recently, a Dutch multicenter research group conducted the NET-QUBIC study in patients with HNC [25]. While smaller in size than the previously mentioned databases, NET-QUBIC provides extensive data on patient and tumor characteristics complemented by a biobank. It also contains multiple patient reported outcome measures (PROMs) focusing on lifestyle and quality of life (QOL), and data on caregivers. In Denmark, the DAHANCA group was established to develop national guidelines targeting patients with HNC [26]. The DAHANCA database registers several basic variables such as symptoms, diagnostic evaluation and primary treatment, and also contains a biobank including tumor tissue, blood samples, and DNA [26]. In addition, a large UK-based clinical cohort study in head and neck cancer called Head and Neck 5000 was set up including data on QoL, blood samples and saliva samples [27]. To monitor and effectively improve high quality integrated care in the Netherlands, the Dutch Head and Neck Audit (DHNA) was set up [28]. This multidisciplinary oncological quality registration includes primary head and neck tumors and aims to monitor, benchmark and find areas for improvement.

**Table 2:** Overview and characteristics of existing head and neck cancer (HNC) databases

	(DAHANCA) [26]	NORDCAN [6, 7]	IKNL [24]	NET-QUBIQ [25]	DHNA [28]	HN5000 study [27, 29]	NCDB [17, 18]	SEER [4, 5]	RONCDOC
**Country of origin**	Denmark	Nordic countries	NL	NL	NL	UK	USA	USA	NL
**Database characteristics**									
Head and cancer specific	yes	no	no	yes	yes	yes	no	no	yes
Pro-/retrospective	pro	retro	retro	pro	pro	pro	retro	retro	both
Hospital (h) or population (p) based	p	p	p	h	p	p	h	p	p
Consecutive	yes	yes	yes	no	yes	no	no	yes	yes
**Tumor characteristics**									
Diagnosis/subsite	x	x	x	x	x	x	x	x	x
TNM classification	x	x	x	x	x	x	x	x	x
** Treatment**	x	x	x	x	x	x	x	x	x
**Sociodemographic data**									
Demographic data	x	x	x	x	x	x	x	x	x
Education/literacy		x		x		x	x		x
Occupation		x		x		x			x
Income				x		x	x		x
**Health and lifestyle**									
Co-morbidity	x			x	x	x	x		x
Smoking/alcohol	x		x	x	x	x			x
Quality of life	x			x	x	x			x
**Follow-up**									
Recurrence	x		x	x	x				x
Mortality	x	x	x	x	x	x	x	x	x
**Biological samples**									
Blood sample	x			x		x			x
Saliva sample				x		x			
Tissue sample	x			x		x			x

*UK* United Kingdom, *USA* United States of America, *NL* The Netherlands

x: variable included in database

### Current application of RONCDOC data

Physicians are often unable to give patients an accurate assessment of their prognosis [30–32], which may result in non-optimal patient counseling and over- or undertreatment [33, 34]. To improve this, more personalized and customized information about patients’ prognosis is needed. Besides TNM-classification, a variety of covariables such as age, primary tumor site, and comorbidity are potential prognostic factors. Recently, our prognostic model OncologIQ [35], which predicts 1- to 10 year overall survival chances among patients with a primary head and neck tumor, was updated using RONDOC data [36–38]. RONCDOC data was also used in other publications reporting on survival rates [39–41].

### Limitations

RONCDOC only includes patients treated at the Erasmus Medical Center Rotterdam. Despite the fact that the largest HNC care center of the Netherlands is located in Rotterdam, this single-center design can result in selection bias. Therefore, we recommend to extend the database nationally. RONCDOC is based on work executed voluntarily by a dedicated team of medical professionals, researchers and students. This team ensures that all data from the IKNL are compared to the data in the EPF. In case of errors, these are analyzed and corrected if applicable. Due to changing members of the RONCDOC study group, one could argue that the interrater-variability may increase. However, the interrater variability was constrained by the validation protocol. Establishing RONCDOC, i.e. analyzing paper patient files and all electronic patients documentaries was time consuming. This was inherent to the used methods to document patient data in the EPF. The majority of data are not structured and therefore not directly usable for database purposes. As such, a different future set up of the EPF may be necessary in order to automatically and efficiently collect data without error, preferably following FAIR guiding principles. The continuous updating and entry of new data remains time-consuming and requires adequate staffing including students and a dedicated senior team (with a data manager) that provides supervision and effectively addresses challenges. Another limitation is the incidence of missing data. However, when the missing (completely) at random assumption is plausible, multiple imputation can be applied to handle the missing data [42, 43].

### Future perspectives

RONCDOC will continue to provide the data that is needed to realize robust and reliable prognostic models for HNC patients, like the recently updated OncologIQ and the development of a prognostic model for the palliative phase [38, 41]. These models could subsequently be used by physicians to improve decision making and optimize individual counseling and empower HNC patients. In order to further personalize counseling on treatment options and survival, a continuous update of RONCDOC including data on QoL is of high importance. In 2013 we developed the Healthcare Monitor (HM), an electronic patient reported outcome (ePRO) based clinical support system, which uses simple and internationally validated questionnaires regarding HNC, measuring physical and psychosocial functioning from day of diagnosis until 5 years after. The ePRO data collected within HM are a great source [44]. Since 2015, the HM is structurally embedded in our care for HNC patients. Repeated measurement data on QoL, psychosocial and physical symptoms from a consecutive cohort will enable valid and reliable predictions of QoL and morbidity in relation to survival.

At present, we are providing steps to connect the currently retrospective RONCDOC database with the prospective HM data and an automated input of clinical data from the EPF and the IKNL. Currently, data from 2017 are included working towards this prospective set-up of RONCDOC. The RONCDOC infrastructure will facilitate future fundamental and clinical studies, by bringing all data together instead of setting up data collection and processing for each project separately. This will also speed up translation of the results to the clinical practice. As for the tissue collection: future efforts will focus on expanding the inventory of histopathological characteristics and tissue sampling to more anatomical localizations within the head and neck region.

## Conclusions

With RONCDOC, we have established a consecutive, high-quality data warehouse for HNC. This paper outlines the necessary steps to establish such a data warehouse, offering a blue print for other consortia. More accurate, valid and multidisciplinary data can make important contributions to future interdisciplinary research and patient care in the field of HNC.

## Supplementary Information

Supplementary Material 1.

## Data Availability

The data that support the findings of this study are available from the RONCDOC consortium but restrictions apply to the availability of these data, which were used under license for the current study, and so are not publicly available. Data are however available from the authors upon reasonable request and with permission of the RONCDOC consortium.

## Abbreviations

AJCC: American Joint Committee on Cancer
CCI: Charlson comorbidity index
DAHANCA: Danish Head and Neck Cancer
DHNA: Dutch Head and Neck Audit
ECOG: Eastern Cooperative Oncology Group
EPF: Electronic patient file
ePRO: Electronic patient reported outcome
FAIR: Findability, Accessibility, Interoperability and Reusability
FFPE: Formalin-fixed paraffin-embedded
Gemstracker: GEneric Medical Survey Tracker
HE: Hematoxylin and eosin stain
HM: Healthcare Monitor
HNC: Head and neck cancer
HPV: Human papilloma virus
IKNL: Netherlands Comprehensive Cancer Organization
NCDB: National Cancer Database
NCR: Netherlands Cancer Registry
NET-QUBIC: NETherlands Quality of Life and Biomedical Cohort study
NPCR: National Program of Cancer Registries
PROMs: Patient reported outcome measures
QoL: Quality of life
RONCDOC: Rotterdam Oncology Documentation
SEER: Surveillance, Epidemiology, and End Results
SPSS: Statistical Package for the Social Sciences
TMA: Tissue Microarrays
U.S.: United States
WHO: World Health Organization
