# Correction: Rotterdam Oncology Documentation (RONCDOC) – a high-quality data warehouse and tissue collection for head and neck cancer

**DOI:** 10.1186/s12885-025-14727-3

**Published:** 2025-07-25

**Authors:** Arta Hoesseini, Emilie A. C. Dronkers, Eveline Dieleman, Maria J. De Herdt, Marjan H. Wieringa, Marie-Louise F. van Velthuysen, Marinella P. J. Oferman, Aniel Sewnaik, Dominiek Monserez, Stijn Keereweer, Jose A. U. Hardillo, Robert Jan Baatenburg de Jong

**Affiliations:** 1 Department of Otorhinolaryngology and Head and Neck Surgery, Erasmus MC Cancer Institute, Erasmus University Medical Center, Dr. Molewaterplein 40, Rotterdam, 3015 GD The Netherlands; 2 Department of Otorhinolaryngology and Head and Neck Surgery, Leiden University Medical Centre, Leiden, The Netherlands; 3 Department of Pathology, Erasmus MC, Erasmus University Medical Center, Rotterdam, The Netherlands; 4 Department of Education and Research, Elisabeth TweeSteden Hospital (ETZ), Tilburg, The Netherlands

**Correction: Cancer 25**,** 778 (2025)**

**https://doi.org/10.1186/s12885-025-14100-4**

Following publication of the original article [1], the authors identified an error in Table 1, whereby the heading row was omitted. The Marital status category is also incorrect and should read as "Married / in a durable relationship or single / widowed" instead of "Married in a durable relationship or single/widowed".

The corrected Table 1 is given below:

**Table:** 

**Variable**	**Category**
**Patient**	
Sex	Female/male
Date of birth	Date
Deceased	Yes/no
Final date of follow-up/date of death	Date
Cause of death	Due to HNC/not due to HNC/unknown/not applicable
Smoking	Yes/no/former
Pack years	
Alcohol consumption	Yes/no/former
No. alcohol units per week	
Adult Comorbidity Evaluation 27	Subscales & total scale
Weight	Kilogram (1 kg = 2.20 pounds)
Weight loss in the past six months	Kilogram (1 kg = 2.20 pounds)
Height	Centimeter (1 cm = 0.39 inches)
Body Mass Index	
WHO performance status (Eastern Cooperative Oncology Group score)	0/1/2/3/4
Anemia	Yes/no, cutoff male < 8.5 mmol/L, cutoff female < 7.5 mmol/L (1 mmol/L = 1.61 g/dL)
Heart valve disease	Yes/no
Marital status	Married / in a durable relationship or single / widowed
Prior malignancy	Yes/no
Localization prior malignancy	Lung/breast/bowel/prostate/hematologic/head and neck/other
Year of diagnosis of prior malignancy	Year
Treatment for prior malignancy?	Yes/no
Type of treatment for prior malignancy	None/radiotherapy/chemoradiation/surgery/surgery & radiotherapy/chemoradiation & surgery/chemotherapy/surgery & chemotherapy
**Tumor**	
Date of diagnosis	Date
Tumor no in chronologic order	1, 2, 3, 4, 5, etc
Primary or recurrent tumor	First primary/second primary/etc First recurrence/second recurrence/etc
Recurrence related to	First primary/second primary/etc
Synchronous/metachronous tumor	Not applicable/synchronous/metachronous
Tumor topography	Lip/oral cavity/oropharynx/hypopharynx/pharynx NOS^a^/glottic larynx/supraglottic larynx/subglottic larynx/larynx NOS^a^/nasopharynx/unknown primary/salivary glands/nasal cavity/middle ear/sinus/thyroid/skin
Site of tumor	Left/right/middle/bilateral
cTNM	According to the TNM- 7
pTNM	According to the TNM- 7
Tumor morphology	Squamous cell carcinoma/adenocarcinoma/etc
Treatment intention	Curative/palliative because of HNC/palliative because of another tumor/refusal of curative treatment/died before completing diagnostics/unknown
Retropharyngeal nodes	Yes/no
HPV-status (p16 immunoreactivity)	Yes/no
**Treatment**	
Treatment intention	Curative/palliative because of HNC/palliative because of another tumor/refusal of curative treatment/died before completing diagnostics/unknown
Did the patient receive treatment?	Yes/no (patient related)/no (physician related)/died before start treatment
Treatment according to protocol?	Yes/no (patient related)/no (physician related)/died before start treatment
Treatment type no. 1^b^	None/radiotherapy/chemotherapy/surgery with lymph node dissection/surgery without lymph node dissection/lymph node dissection/other
Start date of treatment no. 1	Date
End date of treatment no. 1	Date
Was treatment no. 1 completed?	Yes/no/not applicable/unknown
**Pathology**	
Pathology specimen ID	
Pathology specimen date	Date
Pathology specimen type	Biopsy/resection/etc
Pathology specimen characteristics	Tumor site & location/tumor diameter/no. pathological lymph nodes affected (side & level)/diameter of metastasis in mm (if RLNMs are present)/extranodal extension (if RLNMS are present)/depth of invasion (according to 8 th ed AJCC)/resection margin status/WPOI (intended according to 8 th ed AJCC)/invasion pattern/perineural invasion/lymphovascular invasion/histological grade/tumor infiltrating lymphocytes (intended according to 8 th ed AJCC)

Several free text fields were added between variables (not included in this table). A complete overview of all variables can be found in the data dictionary and data entry protocol

^a^Not Otherwise Specified

^b^For each treatment type a separate column was completed on type, start date, end date and completion (no. 1, no. 2, etc.)

The original article [1] has been corrected.
