# Supplementary material for: Rotterdam Oncology Documentation (RONCDOC) – a high-quality data warehouse and tissue collection for head and neck cancer
Source: BMC Cancer. 2025 Apr 25;25:778. doi: 10.1186/s12885-025-14100-4 (PMC12032767; doi:10.1186/s12885-025-14100-4)
Supplement: Supplementary file 1 — Supplementary Material 1. [file 12885_2025_14100_MOESM1_ESM.docx]

**Complete data dictionary and data entry protocol**

**Introduction**

Patient diagnosed with and/or treated for a head and neck tumor in the Erasmus Medical Center Cancer Institute, Rotterdam from the 1st of January 2006 until the 31st of December 2013 have been included in this database. Data collected in the Netherlands Cancer Registry (NCR) of these patients were obtained from the Netherlands comprehensive cancer organization (IKNL) and merged with automatically generated data from the electronic patient file (EPF). These data are manually checked using the EPF. Also, additional variables are added to the database which can only be found in the EPF. For example, smoking behavior, prior malignancy and comorbidity (ACE-27).

In the EPF patient most data be found in the reference tab (‘’naslag’’), basic information (basisgegevens), correspondence (correspondentie), tumor board files (‘’RWHHT formulier’’) and multimedia tab. This manual describes how the data should be scored and entered. Each student has their own code, allowing us to track who entered the data for a specific patient.

The database consists of four main topics:

1. General variables
2. Tumor variables
3. Treatment variables
4. Additional variables like smoking, drinking, ACE-27

Each variable that is included in these topics has 3 separate variables types, namely

1. result from the IKNL database (IKNL_*variablename*)
2. results from the EPF (EMC_*variablename*)
3. corrected result in the case 1) and 2) differ, which is entered after consulting the EPF (*variablename*_cor)

For some variables, there is no EPF data (e.g., pTNM), and for others, there is no IKNL data (e.g., tumor type). In such cases, these variables are assessed in the EPF and filled in in ‘’3) corrected results’’.

Except for topic 1 (General information), all variables are assessed again for each new HNC tumor. In other words: if the patient has a second primary tumor or a recurrence, all variables of topic 2 until 4 are again assessed at the time of diagnosis of the new tumor (‘’incidentiedatum’’).

**Tab 1 General variables**

This tab contains information about the patient's identity and their number in the database. Examples of information found under this tab include the patient ID, the registration number in the IKNL database, gender, date of birth, and whether the patient is still alive.

**Variables** (database label including tumor no.)

**Study ID** (Respondent ID)

**Student number** (Datacontrole) (Datacontroleother)

Each research student has his own number

**Patient ID EPF** (patient_id)

**Registration number IKNL** (keyNKR)

**Sex** (Geslacht)

Male / female

**Date of birth** (Geboortedatum)

dd/mm/jjjj

**Deceased** (Overleden)

Died / alive

**Final date of follow-up / date of death** (FUdatum)

dd/mm/jjjj

If the EPF follow-up date is later than the IKNL date, enter the EPF date in the correction field. If the patient is still alive always enter the last contact date from the EPF (physically or by phone). Future outpatient visits are not included.

**Cause of death** (overlijdensreden)

Due to HNC / not due to HNC / unknown / not applicable

**Additional notes** (Opmalg)

Free text field.

If you have any comments or observations regarding Tab 1, you can you can address them here. Also, make sure to log these comments and discuss them if necessary.

**Tab 2 Tumor variables**

All variables in this tab are assessed again for each new HNC tumor. In other words: if the patient has a second primary tumor or a recurrent tumor, all variables of topic 2 until 4 are again assessed at the time of diagnosis of the new tumor.

**Variables** (database label including tumor no.)

**Tumor no. in chronologic order** (tumornr1)

1, 2, 3, 4, 5, etc. starting from the index tumor, which refers to the first head and neck tumor diagnosed on or after January 1, 2006. Head and neck tumors diagnosed prior to the index tumor are documented under "prior malignancy," as detailed in Tab 4. If after the index tumor a recurrence is diagnosed this is labeled as no. 2 etc.

**Date of diagnosis** (Diagdat_tum1)

dd/mm/jjjj

The date on which the biopsies diagnosing HNC were conducted (sometimes in another hospital).

If histology or cytology results are unavailable, the next best option is used, such as the date of the CT or MRI, followed by the clinical diagnosis. In that case add a comment in the next variable ‘’Opmincidat’’. If only the year of diagnosis is known, use January 1st of that year as the date of incidence.

**Additional notes date of diagnosis** (Opmincidat1)

Free text field.

If you have any comments or observations regarding the date of diagnosis, you can you can address them here.

**Primary or recurrent tumor** (Aardtumor1)

First primary / second primary / etc.

First recurrence / second recurrence / etc.

**Recurrence related to** (TUMBEHORBIJ2)

First primary / second primary / etc.

Only applicable to recurrent tumors.

**Site of tumor** (Zijde1)

Left / right / middle / bilateral

In the case of a metastasis, the side of the primary tumor is entered.

**Synchronous / metachronous tumor** (synchrmetachr2)

Not applicable / synchronous / metachronous

Synchronous: within 6 months of diagnosis of the index tumor.

Metachronous: after 6 months of diagnosis of the index tumor.

**Tumor morphology** (Morf1)

See codes in the addendum. These codes were converted into squamous cell carcinoma, adenocarcinoma etc.

**Tumor topography** (Topog1)

See also codes in the addendum. These codes were converted into the following tumor locations:

Lip / oral cavity / oropharynx / hypopharynx / pharynx NOS* / glottic larynx / supraglottic larynx / subglottic larynx / larynx NOS* / nasopharynx / unknown primary / salivary glands / nasal cavity / middle ear / sinus / thyroid / skin

*Not Otherwise Specified.

**cT** (cT_1)

According to the TNM-7.

**cN** (cN_1)

According to the TNM-7.

**cM** (cM_1)

According to the TNM-7.

**pT** (pT_1)

According to the TNM-7.

**pN** (pN_1)

According to the TNM-7.

**pM** (pM_1)

According to the TNM-7.

**HPV** (P16_status1)

Yes / no (p16 immunoreactivity)

**Retropharyngeal nodes** (RFklier1)

Yes / no

**Additional notes** (OPMERKING1)

Free text field

If you have any comments or observations regarding the tumor information tab, you can you can address them here. Also, make sure to log these comments and discuss them if necessary.

**Tab 3 Treatment variables**

**Variables** (database label including tumor no.)

**Treatment intention** (Beh_Int_1)

Curative / palliative because of HNC / palliative because of another tumor / refusal of curative treatment / died before completing diagnostics / unknown

**Did the patient receive treatment** (Beh_gehad1)

Yes / no (patient related) / no (physician related) / died before start treatment

**Treatment according to protocol** (Protocol_JN1)

Yes / no (patient related) / no (physician related) / died before start treatment

**Treatment type no. 1** (Therap11)

None / radiotherapy / chemotherapy / surgery with lymph node dissection / surgery without lymph node dissection / lymph node dissection / other

For each treatment type a separate column should be completed on type, start date, end date and completion (no. 1, no. 2, etc.). Chemoradiation is recorded as two separate treatments. Treatments should be documented in the order of administration, with the treatment that started first being documented first. Laser surgery can Photodynamic therapy (PDT) can be classified under radiotherapy. Laser surgery is classified under surgery.

**Start date of treatment no. 1** (Therap11_dat)

dd/mm/jjjj

If the exact start date is unknown but the month is known, enter the 15th of the month.

**End date of treatment no. 1** (Therap_einddat1)

dd/mm/jjjj

If the exact start date is unknown but the month is known, enter the 15th of the month.

**Was treatment no. 1 completed** (Therap_af1)

Yes / no / unknown / not applicable

**Additional notes** (Opmther1)

Free text field

If you have any comments or observations regarding the treatment tab, you can address them here. Also, make sure to log these comments and discuss them if necessary.

**Tab 4 Additional variables like smoking, drinking, ACE-27**

**Variables** (database label including tumor no.)

**Smoking** (Roken1)

Yes / no / former

When "yes / former" is selected, information is requested on the number of pack-years, units per day, and the number of years the patient has smoked. If a patient has stopped smoking for ≥ 3 months, he or she is considered as a former smoker. If the patient is a former smoker, the year in which they quit is also requested.

**Pack years** (RookPY1)

The cumulative quantity of smoking is defined in pack-years in which one pack year is equal to one pack of 20 cigarettes smoked per day for one year.

Calculating the number of cigarettes:¹ˉ²

1 cigarette = 1 g tobacco

1 cigar = 4 g tobacco = 4 cigarettes

1 pipe = 3.5 g tobacco = 3.5 cigarettes

1 shag = 0.7 g tobacco (on average, 45 g of tobacco = 65 roll-your-own cigarettes)

1 pouch of shag per day is equivalent to 2.275 packs of cigarettes/day

When different quantities are found in the EPF, the highest quantity is used.

If a range is given, such as 15-20 cigarettes per day, take the average - in this case, 18 cigarettes per day.

If it is stated that someone has smoked their entire life, assume they started at the age of 18, unless other information is provided.

**Number of tobacco units per day** (rookhoeveel1)

Number of tobacco units per day, 1 unit = 1 gram.

**No. of years smoked** (rookduur1)

Total number of years smoked.

**Year stopped smoking** (rookstop1)

The year in which the patient quit smoking.

**Alcohol consumption** (Alcohol1)

Yes / no / former

If a patient had stopped drinking for ≥ 6 months he or she is considered as a former drinker.

**Number of alcohol units per week** (Alcoholeenheden1)

No. of alcohol units per week are scored according to a standardized list: one unit (E), or 10 grams of alcohol is equivalent to 12.5 milliliters of pure ethanol.

When ‘’yes / former" is selected, information is requested on the number of units per week, number of years drinking, and the year the patient stopped drinking.

Calculating the number of units:³ˉ⁴

1E alcohol = 10 g of 12.5 ml pure ethanol

1E glass of beer 5% = 250 ml, 1 bottle/can of beer 5% 330 ml = 1.3E

1E glass of wine 12% = 105 ml, 1 bottle of wine 12% 750 ml = 7E

1E port 19%, 1 bottle of port 19% 750 ml = 11E
1E gin 35% = 35 ml, 1 bottle of gin 35% 750 ml = 21E

1E vodka/whiskey 40% = 30 ml, 1 bottle of vodka/whiskey 40% 750 ml = 24E

‘’Social drinking’’ is considered as 2 units/week, unless more information is provided.

‘’Sporadic’’ alcohol consumption is equivalent to no alcohol consumption (less than 1 unit/week), unless

more information is provided.

**No. of years consuming alcohol** (Drinktinjaren1)

Total number of years drinking alcohol.

**Year stopped consuming alcohol** (drinkstop1)

The year in which the patient stopped consuming alcohol.

**Additional notes on smoking and/or consuming alcohol** (Opmrookdrink1)

Free text field

**Adult Comorbidity Evaluation 27** (ACE27.1)

Subscales (ACE1.1), (ACE1.2) etc. and total scale (ACE27.1)

See separate manual.

**Height** (Lengte1)

Centimeter (1 cm = 0.39 inches)

**Weight** (Gewicht1)

Kilogram (1 kilogram = 2.20 pound)

**Body Mass Index** (BMI1)

Is automatically generated.

**Weight loss in the past six months** (gewichtsverlies1)

Weight loss in kilograms (kg) is defined as weight loss in the six months before diagnosis. If there are multiple measurements, take the average.

Kilogram (1 kilogram = 2.20 pound)

**Prior malignancy** (Priormalign1)

Yes / no

If your index tumor is a recurrence of the first primary head and neck tumor, record the details of the first primary tumor under "prior malignancy."

**Localization prior malignancy** (Priorlocal1) and (Priorlocal1other)

Lung / breast / bowel / prostate / hematologic / head and neck / other

In you chose "other", a free text field will appear. Here you can specify which malignancy/malignancies were previously diagnosed in the patient.

**Year of diagnosis of prior malignancy** (Priorjaar1)

Year

**Treatment for prior malignancy?** (Priorbeh1)

Yes / no

**Type of treatment for prior malignancy** (Priorbehtype1)

None / radiotherapy / chemoradiation / surgery / surgery & radiotherapy / chemoradiation & surgery / chemotherapy / surgery & chemotherapy

**WHO performance status**  (WHOstat1)

The World Health Organization (WHO) performance status, also known as the Eastern Cooperative Oncology Group (ECOG) score, is scored according to the classification published by Oken et al.⁵

0 = Fully active, able to carry on all pre-disease performance without restriction.

1 = Restricted in physically strenuous activity but ambulatory and able to carry out work of a light or sedentary nature, e.g., light house work, office work.

2 = Ambulatory and capable of all selfcare but unable to carry out any work activities. Up and about more than 50% of waking hours.

3 = Capable of only limited selfcare, confined to bed or chair more than 50% of waking hours.

4 = Completely disabled. Cannot carry on any selfcare. Totally confined to bed or chair.

In case of doubt about the WHO grade, choose the least severe option. If a patients’ classification falls between WHO 2 and 3, select 2.

In the case the Karnofsky index is used, apply the following:

Karnofsky 80 - 100 equals WHO 0 - 1

Karnofsky 60 - 70 equals WHO 2

Karnofsky 10 - 50 equals WHO 3 – 4

**Anemia** (Anemie1)

Yes / no

Hemoglobin in mmol/L (1 mmol/L = 1.61 g/dL). Cutoff male < 8.5 mmol/L, cutoff female < 7.5.

Presence of anemia at the time of diagnosis or before the start of treatment

**Heart valve disease** (Hartklep1)

Yes / no

Presence of heart valve disease at the time of diagnosis or before the start of treatment

**Marital status** (Alleen1_def)

Yes / no

Is the patient single?

Single or widowed = yes, married or in a durable relationship = no.

After completing all variables, the list is submitted. The 'resume later' option should only be used in exceptional cases, such as for complex patients who need to be discussed. You can always modify the answers later using the 'redo, keep answers' button. When you click 'resume later' after entering the answers, the responses are stored in a shadow file and not in the actual database.

¹ Wood, D. M., Mould, M. G., Ong, S. B. Y., & Baker, E. H. (2005). “Pack year” smoking histories: what about patients who use loose tobacco?. Tobacco control, 14(2), 141-142.

² Wyss, A., Hashibe, M., Chuang, S. C., Lee, Y. C. A., Zhang, Z. F., Yu, G. P., ... & Olshan, A. F. (2013). Cigarette, cigar, and pipe smoking and the risk of head and neck cancers: pooled analysis in the International Head and Neck Cancer Epidemiology Consortium. American journal of epidemiology, 178(5), 679-690.

³ Lemmens, P. H. (1994). The alcohol content of self‐report and ‘standard’drinks. Addiction, 89(5), 593-601.

⁴ Science and Technology Committee. (2012). Alcohol Guidelines: Eleventh Report of Session 2010-12. House of Commons, London: The Stationery Office Limited.

⁵ Oken MM, Creech RH, Tormey DC, Horton J, Davis TE, McFadden ET, et al. Toxicity and response criteria of the Eastern Cooperative Oncology Group. Am J Clin Oncol. 1982;5(6):649-55.

**Addendum**

| ICD-0-3 MORPHOLOGY CODES | | INCLUSION |
| --- | --- | --- |
| 8070/2 | Squamous cell carcinoma in situ, NOS | Control tissue |
| 8070/3 | Squamous cell carcinoma, NOS | Yes |
| 8070/6 | Squamous cell carcinoma, metastatic, NOS | No |
| 8071/3 | Squamous cell carcinoma, keratinizing, NOS | Yes |
| 8071/6 | Squamous cell carcinoma, keratinizing, metastatic, NOS | No |
| 8072/3 | Squamous cell carcinoma, large cell, nonkeratinizing, NOS | Yes |
| 8072/6 | Squamous cell carcinoma, large cell, nonkeratinizing, metastatic, NOS | No |
| 8073/3 | Squamous cell carcinoma, small cell, nonkeratinizing | Yes |
| 8073/6 | Squamous cell carcinoma, small cell, nonkeratinizing, metastatic | No |
| 8074/3 | Squamous cell carcinoma, spindle cell | Yes |
| 8074/6 | Squamous cell carcinoma, spindle cell, metastatic | No |
| 8075/3 | Squamous cell carcinoma, adenoid | Yes |
| 8075/6 | Squamous cell carcinoma, adenoid, metastatic | No |
| 8076/2 | Squamous cell carcinoma in situ with questionable stromal invasion | Control tissue |
| 8076/3 | Squamous cell carcinoma, microinvasive | Control tissue |
| 8076/6 | Squamous cell carcinoma, microinvasive, metastatic | No |
| 8077/2 | Squamous intraepithelial neoplasia, high grade | Control tissue |
| 8078/3 | Squamous cell carcinoma with horn formation | Yes |
| 8078/6 | Squamous cell carcinoma with horn formation, metastatic | No |

|  |  |  |  |  |  |  |
| --- | --- | --- | --- | --- | --- | --- |
| TOPOGRAFIECODE | INCLUSION | Anatomical localisation | Regions | ICD-O-3 | Site | Related term |
| C000 | No | Oral cavity | Lip | C00.0 | Lip | External upper lip |
| C001 | No | Oral cavity | Lip | C00.1 | Lip | External lower lip |
| C002 | No | Oral cavity | Lip | C00.2 | Lip | External lip, NOS |
| C003 | Yes | Oral cavity | Lip | C00.3 | Lip | Mucosa of upper lip |
| C004 | Yes | Oral cavity | Lip | C00.4 | Lip | Mucosa of lower lip |
| C005 | Yes | Oral cavity | Lip | C00.5 | Lip | Mucosa of lip, NOS |
| C006 | No | Oral cavity | Lip | C00.6 | Lip | Commissure of lip |
| C008 | No | Oral cavity | Lip | C00.8 | Lip | Overlapping lesion of lip |
| C009 | No | Oral cavity | Lip | C00.9 | Lip | Lip, NOS (excludes Skin of lip C44.0) |
| C019 | Yes | Pharynx | Oropharynx | C01.9 | Base of tongue | Base of tongue, NOS |
| C020 | Yes | Oral cavity | Oral cavity | C02.0 | Other and unspecified parts of tongue | Dorsal surface of tongue, NOS |
| C021 | Yes | Oral cavity | Oral cavity | C02.1 | Other and unspecified parts of tongue | Border of tongue |
| C022 | Yes | Oral cavity | Oral cavity | C02.2 | Other and unspecified parts of tongue | Ventral surface of tongue, NOS |
| C023 | Yes | Oral cavity | Oral cavity | C02.3 | Other and unspecified parts of tongue | Anterior 2/3 of tongue, NOS |
| C024 | Yes | Pharynx | Oropharynx | C02.4 | Other and unspecified parts of tongue | Lingual tonsil |
| C028 | Yes | Oral cavity | Oral cavity | C02.8 | Other and unspecified parts of tongue | Overlapping lesion of tongue |
| C029 | Yes | Oral cavity | Oral cavity | C02.9 | Other and unspecified parts of tongue | Tongue, NOS |
| C030 | Yes | Oral cavity | Oral cavity | C03.0 | Gum | Upper gum |
| C031 | Yes | Oral cavity | Oral cavity | C03.1 | Gum | Lower gum |
| C039 | Yes | Oral cavity | Oral cavity | C03.9 | Gum | Gum, NOS |
| C040 | Yes | Oral cavity | Oral cavity | C04.0 | Floor of mouth | Anterior floor of mouth |
| C041 | Yes | Oral cavity | Oral cavity | C04.1 | Floor of mouth | Lateral floor of mouth |
| C048 | Yes | Oral cavity | Oral cavity | C04.8 | Floor of mouth | Overlapping lesion of floor of mouth |
| C049 | Yes | Oral cavity | Oral cavity | C04.9 | Floor of mouth | Floor of mouth, NOS |
| C050 | Yes | Oral cavity | Oral cavity | C05.0 | Palate | Hard palate |
| C051 | Yes | Pharynx | Oropharynx | C05.1 | Palate | Soft palate, NOS (excludes nasopharyngeal surface of soft palate C11.3) |
| C052 | Yes | Pharynx | Oropharynx | C05.2 | Palate | Uvula |
| C058 | Yes | Oral cavity | Oral cavity | C05.8 | Palate | Overlapping lesion of palate |
| C059 | Yes | Oral cavity | Oral cavity | C05.9 | Palate | Palate, NOS |
| C060 | Yes | Oral cavity | Oral cavity | C06.0 | Other and unspecified parts of mouth | Cheek mucosa |
| C061 | Yes | Oral cavity | Oral cavity | C06.1 | Other and unspecified parts of mouth | Vestibule of mouth |
| C062 | Yes | Oral cavity | Oral cavity | C06.2 | Other and unspecified parts of mouth | Retromolar area |
| C068 | Yes | Oral cavity | Oral cavity | C06.8 | Other and unspecified parts of mouth | Overlapping lesion of other and unspecified parts of mouth |
| C069 | Yes | Oral cavity | Oral cavity | C06.9 | Other and unspecified parts of mouth | Mouth, NOS |
| C079 | No | Major salivary glands | Major salivary glands | C07.9 | Parotid gland | *Parotid gland |
| C080 | No | Major salivary glands | Major salivary glands | C08.0 | Other and unspecified major salivary gland | *Submandibular gland |
| C081 | No | Major salivary glands | Major salivary glands | C08.1 | Other and unspecified major salivary gland | *Sublingual gland |
| C088 | No | Major salivary glands | Major salivary glands | C08.8 | Other and unspecified major salivary gland | Overlapping lesion of major salivary glands |
| C089 | No | Major salivary glands | Major salivary glands | C08.9 | Other and unspecified major salivary gland | *Major salivary gland, NOS (excludes minor salivary gland, NOS C06.9 |
| C090 | Yes | Pharynx | Oropharynx | C09.0 | Tonsil | *Tonsillar fossa |
| C091 | Yes | Pharynx | Oropharynx | C09.1 | Tonsil | *Tonsillar pillar |
| C098 | Yes | Pharynx | Oropharynx | C09.8 | Tonsil | Overlapping lesion of tonsil |
| C099 | Yes | Pharynx | Oropharynx | C09.9 | Tonsil | *Tonsil, NOS (excludes lingual tonsil C02.4 and pharyngeal tonsil C11.1) |
| C100 | Yes | Pharynx | Oropharynx | C10.0 | Oropharynx | Vallecula |
| C101 | Yes | Pharynx | Oropharynx | C10.1 | Oropharynx | Anterior surface of epiglottis |
| C102 | Yes | Pharynx | Oropharynx | C10.2 | Oropharynx | Lateral wall of oropharynx |
| C103 | Yes | Pharynx | Oropharynx | C10.3 | Oropharynx | Posterior wall of oropharynx |
| C104 | Yes | Pharynx | Oropharynx | C10.4 | Oropharynx | Branchial cleft (site of neoplasm) |
| C108 | Yes | Pharynx | Oropharynx | C10.8 | Oropharynx | Overlapping lesion of oropharynx |
| C109 | Yes | Pharynx | Oropharynx | C10.9 | Oropharynx | Oropharynx, NOS |
| C110 | No | Pharynx | Nasopharynx | C11.0 | Nasopharynx | Superior wall of nasopharynx |
| C111 | No | Pharynx | Nasopharynx | C11.1 | Nasopharynx | Posterior wall of nasopharynx |
| C112 | No | Pharynx | Nasopharynx | C11.2 | Nasopharynx | Lateral wall of nasopharynx |
| C113 | No | Pharynx | Nasopharynx | C11.3 | Nasopharynx | Anterior wall of nasopharynx |
| C118 | No | Pharynx | Nasopharynx | C11.8 | Nasopharynx | Overlapping lesion of nasopharynx |
| C119 | No | Pharynx | Nasopharynx | C11.9 | Nasopharynx | Nasopharynx, NOS |
| C129 | Yes | Pharynx | Hypopharynx | C12.9 | Pyriform sinus | Pyriform sinus |
| C130 | Yes | Pharynx | Hypopharynx | C13.0 | Hypopharynx | Postcricoid region |
| C131 | Yes | Pharynx | Hypopharynx | C13.1 | Hypopharynx | Hypopharyngeal aspect of aryepiglottic fold, NOS (excludes laryngeal aspect of aryepiglottic fold C32.1) |
| C132 | Yes | Pharynx | Hypopharynx | C13.2 | Hypopharynx | Posterior wall of hypopharynx |
| C138 | Yes | Pharynx | Hypopharynx | C13.8 | Hypopharynx | Overlapping lesion of hypopharynx |
| C139 | Yes | Pharynx | Hypopharynx | C13.9 | Hypopharynx | Hypopharynx, NOS |
| C140 | No | Pharynx | NOS | C14.0 | Other and ill-defined sites in lip, oral cavity and pharynx | Pharynx, NOS |
| C142 | Yes | Pharynx | Oropharynx | C14.2 | Oropharynx | Waldeyer's ring |
| C148 | No | NOS | NOS | C14.8 | Other and ill-defined sites in lip, oral cavity and pharynx | Overlapping lesion of lip, oral cavity and pharynx |
| C300 | No | Nasal cavity | Nasal cavity | C30.0 | Nasal cavity | *Nasal cavity (excludes Nose, NOS C76.0) |
| C301 | No | Middle ear | Middle ear | C30.1 | Middle ear | Middle ear |
| C310 | No | Paranasal sinus | Paranasal sinus | C31.0 | Accessory sinuses | *Maxillary sinus |
| C311 | No | Paranasal sinus | Paranasal sinus | C31.1 | Accessory sinuses | Ethmoid sinus |
| C312 | No | Paranasal sinus | Paranasal sinus | C31.2 | Accessory sinuses | *Frontal sinus |
| C313 | No | Paranasal sinus | Paranasal sinus | C31.3 | Accessory sinuses | Sphenoid sinus |
| C318 | No | Paranasal sinus | Paranasal sinus | C31.8 | Accessory sinuses | Overlapping lesion of accessory sinuses |
| C319 | No | Paranasal sinus | Paranasal sinus | C31.9 | Accessory sinuses | Accessory sinus, NOS |
| C320 | Yes | Larynx | Glottis | C32.0 | Larynx | Glottis |
| C321 | Yes | Larynx | Supraglottis | C32.1 | Larynx | Supraglottis |
| C322 | No | Larynx | Subglottis | C32.2 | Larynx | Subglottis |
| C323 | No | Larynx | NOS | C32.3 | Larynx | Laryngeal cartilage |
| C328 | No | Larynx | NOS | C32.8 | Larynx | Overlapping lesion of larynx |
| C329 | No | Larynx | NOS | C32.9 | Larynx | Larynx, NOS |
| C739 | No | Thyroid | Thyroid gland | C73.9 | Thyroid gland | Thyroid gland |

|  |
| --- |
| *Specific sub sites of these organs which are considered lateral sites are indicated with an asterisk (*). |
|  |
